# Metabolic and immune functions of the hemolymph and fat body in *Hermetia illucens* (Diptera: Stratiomyidae) under pathogen challenge

**DOI:** 10.1093/jisesa/ieaf074

**Published:** 2025-10-11

**Authors:** Neta Herman, Tzach Vitenberg, Itai Opatovsky

**Affiliations:** Laboratory of Insect Nutrition and Metabolism, Department of Nutrition and Natural Products, MIGAL—Galilee Research Centre, Kiryat Shmona, Israel; Department of Animal Science, Faculty of Sciences and Technology, Tel-Hai Academic College, Upper Galilee, Israel; Laboratory of Insect Nutrition and Metabolism, Department of Nutrition and Natural Products, MIGAL—Galilee Research Centre, Kiryat Shmona, Israel; Department of Animal Science, Faculty of Sciences and Technology, Tel-Hai Academic College, Upper Galilee, Israel; Laboratory of Insect Nutrition and Metabolism, Department of Nutrition and Natural Products, MIGAL—Galilee Research Centre, Kiryat Shmona, Israel; Department of Animal Science, Faculty of Sciences and Technology, Tel-Hai Academic College, Upper Galilee, Israel

**Keywords:** black soldier fly, RNA-seq, proteomic, hexamerins, odorant-binding

## Abstract

The hemolymph and fat body tissues are essential for energy storage, metabolism, and immune defense in larval *Hermetia illucens* (L.). We analyzed the diverse proteins and genes expressed in the hemolymph and fat body of larval *H. illucens* in both naïve and pathogen-challenged conditions using the entomopathogenic fungus, *Beauveria bassiana* (Siemaszko). Notably, odorant-binding-like and cuticle-related proteins were abundant, with pathogen exposure leading to significant regulatory changes, highlighting their potential roles in immune defense. We examined the genes associated with key metabolic pathways in *H. illucens*, such as lipid storage, protein storage, fatty acid metabolism, and gluconeogenesis. Interestingly, eight different storage protein-encoding genes (hexamerins/larval serum proteins) with considerable sequence differences were expressed in the fat body. Exposure to *B. bassiana* resulted in significant downregulation of most of these storage protein-encoding genes, which was correlated with reduced larval body weight and probably fewer energy reserves for pupal development. These findings deepen our understanding of the physiological mechanisms by which larval *H. illucens* responds to pathogens and highlight the vulnerability of key metabolic pathways to stress. This study underscores the importance of the fat body and hemolymph in the metabolic and immune functions of insects, paving the way for future research into the molecular pathways governing their adaptation and resilience.

## Graphical abstract

**Graphical Abstract:**
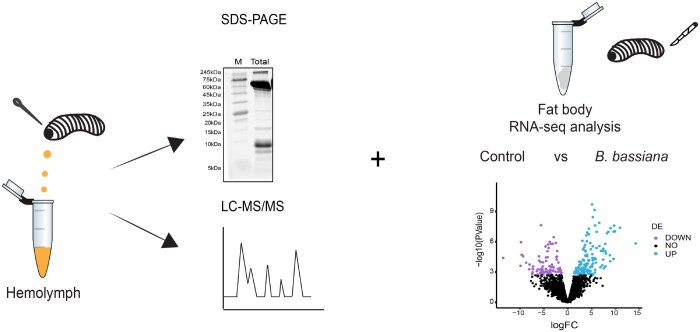

## Introduction

Nutrient transport and storage processes during the larval stage of flies are crucial, as they directly influence larval development, biomass composition, and the fly’s suitability for producing valuable nutrients. Central to these processes are the hemolymph and the fat body, two vital components of insect physiology. The hemolymph, the circulatory fluid in insects, facilitates the transport of nutrients, hormones, and metabolic byproducts, while also providing immune defense against pathogens (Jungreis 1980, Kanost 2009, Blow and Douglas 2019). The fat body, distributed around the internal organs, is the primary site for storing fats, carbohydrates, and proteins. This tissue regulates energy reserves such as fat and glycogen, produces hemolymph proteins and metabolites, and secretes critical proteins such as hexamerins, lipophorins, and vitellogenins essential for growth, metamorphosis, and reproduction (Kilby 1963, Haunerland 1996, Arrese and Soulages 2010, Mani et al 2016).

Hexamerins are large storage proteins comprising six subunits (approximately 80 kDa each) (Telfer and Kunkel 1991). The insect fat body serves as their synthesis site and storage reservoir, with differential expression and localization occurring across differentiated fat body tissues and different stages (Chandrasekar et al. 2008). Following synthesis, hexamerins are secreted into the larval hemolymph where they accumulate to high concentrations. During subsequent developmental transitions, the hexamerins are reabsorbed by the fat body, providing essential amino acids and energy reserves for metamorphosis, adult development, and other physiological processes during non-feeding periods. These proteins also play roles in hormone transport, cuticle formation, and immune defense (Telfer and Kunkel 1991, Chandrasekar et al. 2008, Burmester 2013). In insects, two types of hexamerins, larval serum protein 1 and larval serum protein 2 have been identified, differing in their amino acid composition and aromaticity (Burmester 2013).

Similarly, the fat body manages lipid reserves as cytoplasmic lipid droplets through two lipid storage droplet proteins: lipid storage droplet protein 1, which regulates lipid breakdown, and liquid storage droplet protein 2, which promotes lipid accumulation (Arrese and Soulages 2010). Glucose, stored as glycogen in the fat body, is converted into trehalose, the primary sugar in the hemolymph, which supports energy production and chitin synthesis (Steele 1982, Arrese and Soulages 2010).

The larvae of *Hermetia illucens* (L.) (Diptera: Stratiomyidae), the black soldier fly, efficiently convert diverse organic materials into protein-rich biomass, along with lipids, minerals, and chitin, making them valuable as sustainable animal feed and a resource for biodiesel production, and for applications in biotechnology (Surendra et al. 2020). Therefore, it is crucial to study the core tissues associated with their energy storage and metabolism, such as the hemolymph and fat body. Additionally, *H. illucens* thrives in decaying organic matter, exposing it to a wide range of microbial communities. While antimicrobial peptide production and differential accumulation of metabolites in response to microbial exposure have been studied (Vogel et al. 2018, Opatovsky et al. 2021, Pei et al. 2022, Van Moll et al. 2022, Mani et al. 2024, Herman et al. 2024), other immune-related processes and energy storage in response to pathogens, remain less explored.

We conducted an integrated proteomic and transcriptomic analysis of the *H. illucens* hemolymph and fat body under both naïve and pathogen-challenged conditions using the entomopathogenic fungus, *Beauveria bassiana* (Siemaszko) (Hypocreales: Cordycipitaceae). This fungus penetrates the insects’ cuticle and reaches the insect hemolymph, where it forms yeast-like blastospores that rapidly proliferate, invade internal tissues, and evade the host immune system (Butt et al. 2016, Pedrini 2022). During colonization, *B. bassiana* produces toxic metabolites that suppress the host’s immune response, damage internal tissues and deplete nutrients (Wang et al. 2021). Previous studies have shown that *B. bassiana* infection reduces *H. illucens* larval and adult weight, impairs adult emergence, alters metabolism, and, at high doses, leads to significant mortality and reduced egg production (Lecocq et al. 2021, Mani et al. 2023).

In this study, we examined the diverse array of proteins and RNA expression profiles associated with the hemolymph and fat body in *H. illucens*. We also explore how pathogen exposure modulates the expression of genes related to nutrient reservoirs and metabolism. Our findings provide valuable insights into the metabolic and immune interactions that occur during pathogen stress. This integrative approach enhances our understanding of energy storage and metabolism in insects.

## Materials and Methods

### Larvae Rearing

Larval *H. illucens* were obtained from FreezeM (Nachshonim, Israel) and transferred to a sterile standard Gainesville diet (30% alfalfa, 50% wheat bran, and 20% corn) (Hogsette 1992) to reverse the growth pause induced by the FreezeM technology. Rearing conditions were maintained in a controlled chamber at 27 ± 1.0 °C, with 70 ± 5% RH and a 12 h D:L photoperiod.

### Hemolymph Collection

Hemolymph collection was performed as previously described (Mani et al. 2024). Larvae were reared for 15 d on a sterile standard Gainesville diet, after which larval fifth instar were collected. The larval surfaces were sterilized with 70% EtOH. The hemolymph was collected into a 1.7 ml tube with a few phenylthiourea crystals, from approximately 40 larvae (∼800 µl of hemolymph), by gently puncturing the cuticle in the head region and applying mild pressure. The collected hemolymph was centrifuged at 12,000 g for 10 min to separate the hemocytes, and the supernatant was retained.

### Sodium Dodecyl Sulfate–Polyacrylamide Gel Electrophoresis

A 1.5 µl subsample of the hemolymph sample (∼50 µg) was combined with 4× Laemmli sample buffer (Bio-Rad, Hercules, CA, United States) and water. The mixture was boiled at 95 °C for 7 min, then loaded onto a sodium dodecyl sulfate–polyacrylamide gel electrophoresis (SDS–PAGE) gel with 13% separating and 4% stacking gels (Laemmli 1970). Protein bands were visualized using GelCode Blue Stain Reagent (Thermo Fisher Scientific, Waltham, MA, United States) and the ChemiDoc MP Imaging System (Bio-Rad, Hercules, CA, United States).

### Proteolysis and Liquid Chromatography with Tandem Mass Spectrometry

Proteolysis and liquid chromatography with tandem mass spectrometry (LC–MS/MS) were conducted following previously established protocols (Herman et al. 2024). Protein samples were solubilized in 8.5 M urea, 100 mM ammonium bicarbonate, and 10 mM dithiothreitol, followed by protein quantification using the Bradford assay (Bio-Rad, Hercules, CA, United States). Reduction was performed at 60 °C for 30 min, and modified with 35.2 mM iodoacetamide in 100 mM ammonium bicarbonate at room temperature for 30 min in the dark. Digestion was conducted overnight at 37 °C using modified trypsin (Promega, Madison, WI, United States) in 1.5 M Urea and 17.6 mM ammonium bicarbonate at a 1:50 (M/M) enzyme-to-substrate ratio.

Peptides were analyzed by LC–MS/MS using a Q Exactive HF mass spectrometer (Thermo Fisher Scientific, Waltham, MA, United States) coupled to an easy nLC 1200 system. Peptide separation was achieved on a homemade capillary column (30 cm, 75 µm ID) packed with Reprosil C18-Aqua (Dr Maisch GmbH, Germany). Samples were loaded in solvent A (0.1% formic acid in water) and eluted using a linear gradient of 5–28% solvent B (80% acetonitrile, 0.1% formic acid) over 60 min, followed by a 15-min gradient from 28% to 95% and an additional 15 min at 95% solvent B, with a flow rate of 0.15 μl/min.

Mass spectrometry was performed in positive ion mode (m/z 300–1800), with a resolution of 60,000 for MS1 and 15,000 for MS2. Data acquisition involved repeated full MS scans, followed by high-energy collision dissociation at 27 normalized collision energy of the 18 most abundant ions (charge state > 1) from each MS1 scan. A dynamic exclusion list was enabled with a 20-s exclusion duration to minimize redundant fragmentation.

### Protein Identification and Analysis

Protein identification was performed using Discoverer software (MaxQuant) (Tyanova et al. 2016), searching against two protein datasets derived from the *H. illucens* transcriptome: (i) the UniProt FASTA database of *H. illucens*, including both reviewed and unreviewed sequences (UniProt Consortium, 2023); and (ii) GCF_905115235.1_iHerIll2.2.curated.20191125_protein.faa (National Center for Biotechnology Information [NCBI] genome assembly iHerIll2.2.curated.20191125) (Generalovic et al. 2021). The search protocol was conducted as previously described (Herman et al. 2024). Intensity-based absolute quantification values were employed to estimate protein quantification in the sample. The corresponding genes for all identified protein groups were subjected to functional annotation analysis using the DAVID tool (Huang et al. 2009), with the background list provided by the software.

### RNA-Sequencing Data Collection and Analysis

Data for RNA-sequencing (RNA-seq) were obtained from a previous study (Herman et al. 2024). The dataset included gene count data from the fat body of fifth instar larvae of *H. illucens*, either treated with *B. bassiana* or untreated, with four biological replicates (five larvae in each replicate). To evaluate mRNA expression levels under naïve conditions (untreated samples), FPKM (Fragments Per Kilobase of transcript per Million mapped reads) values were calculated using edgeR. The 1,000 genes with the highest average FPKM values were subjected to functional annotation analysis using the DAVID tool (Huang et al. 2009) with a background list comprising genes with a read count >5 in at least three samples.

Differential expression analysis between *B. bassiana*-treated and untreated groups was conducted using the edgeR (Chen et al. 2025) and RUVseq (Risso et al. 2014) R packages (R Core Team 2023) to account for unwanted variation in the RNA-seq data. Differentially expressed genes were identified based on an FDR (false discovery rate) <0.1 and an absolute fold-change >1.5 as previously performed (Mukherjee et al. 2014, Herman et al. 2022, Rahmé et al. 2025). Upregulated genes (FDR < 0.1 and fold-change > 1.5) and downregulated genes (FDR < 0.1 and fold-change < −1.5) were separately subjected to functional annotation analysis using the DAVID tool (Huang et al. 2009) and the same background list of genes with a read count >5 in at least three samples. We presented the five most significant molecular function terms enriched with an FDR <0.05 separately for upregulated and downregulated genes. An exact binomial test was conducted using R (R Core Team 2023) to evaluate whether the probability of upregulated genes differed from 0.5. Similarly, we tested whether the probability of upregulated genes associated with the cuticle and chitin-binding differed from 0.5 with an exact binomial test.

Multiple sequence alignment analysis of the eight predicted storage proteins was conducted using msa: an R package for multiple sequence alignment (Bodenhofer et al. 2015).

### *B. bassiana* Treatment and Body Weight Measurement of Larval *H. illucens*

Larval *H. illucens* were treated with *B. bassiana* as previously described (Herman et al. 2024). Larvae were reared for five d on a sterile standard Gainesville diet and then divided into two groups: control and *B. bassiana*. Each group comprised of four biological replicates, with each replicate comprising 50 larvae reared on 25 g of the standard Gainesville diet. For the control group, 50 ml sterile water was added, while for the *B. bassiana* treatment, 50 ml of a 10^7^ cells/ml solution of the diluted (11.3%) commercial GHA strain (Rimi, Petah Tikva, Israel) was used. Fungal spores were quantified using a hemocytometer, and the 10^7^ cells/ml solution was prepared by mixing 10 ml commercial strain with 40 ml sterile water.

Ten days after fungal treatment, the average weight of larval fifth instar was measured. Three groups of 10 larvae were weighed for each biological replicate and the average weight was calculated. A *t* test was conducted to compare the means of control and *B. bassiana*-treated groups (*n *= 4 per group), using R with equal variances assumed (R Core Team 2023).

## Results

### Total Protein Content in the Hemolymph of Larval *H. illucens*

**Fig. 1.**
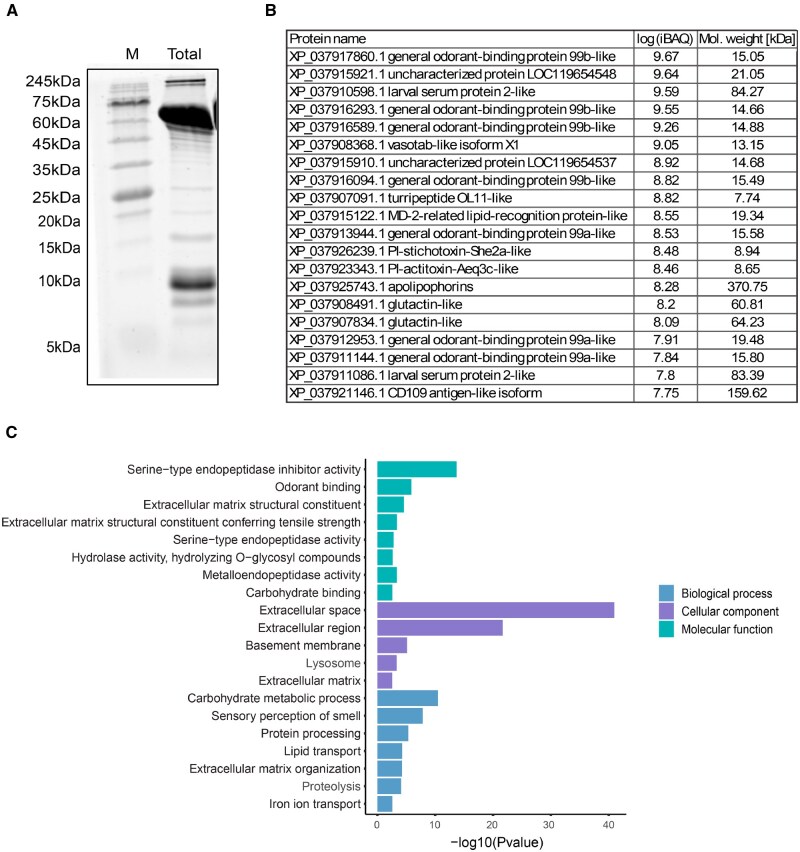
Total proteins in larval hemolymph of *Hermetia illucens*. A) SDS-PAGE analysis of total hemolymph proteins from larval *H. illucens*. B) The 20 most abundant proteins identified through LC-MS/MS analysis of *H. illucens* hemolymph. Quantification is based on intensity-based absolute quantification, with the predicted molecular weight (kDa) derived from protein sequences also presented. C) Significantly enriched gene ontology terms (FDR < 0.05) identified among all proteins from *H. illucens* hemolymph, categorized into biological processes, cellular components, and molecular functions. M, marker lane; Total, total hemolymph proteins.

To analyze the total hemolymph proteins of larval fifth-instar *H. illucens*, we used SDS–PAGE, which revealed several prominent bands. The most dominant bands were observed at approximately 10 kDa, 60–70 kDa, and above 245 kDa (Fig. 1A). To gain deeper insights, we performed LC–MS/MS on the hemolymph, identifying 296 protein groups (Supplementary Table S1). The 20 most abundant proteins, along with their predicted molecular weights, are shown in Fig. 1B. The list primarily includes various types of general odorant-binding protein 99-like proteins. Additionally, it includes storage proteins (larval serum protein 2-like), protease inhibitors (PI-actitoxin-aeq3c-like, PI-stichotoxin-she2a-like, turripeptide OL11-like, and vasotab), lipid transport-associated proteins (apolipophorins), and hemolymph coagulation contributors (glutactin-like).

Gene ontology (GO) enrichment analysis indicated that the 296 proteins were predominantly enriched (FDR < 0.05) in molecular functions such as serine-type endopeptidase inhibitor activity, odorant binding, and extracellular matrix structural constituents, with localization mainly in the extracellular space. The associated biological processes included carbohydrate metabolism, sensory perception of smell, protein processing, and lipid transport (Fig. 1C).

### Gene Expression Profiling in the Fat Body of Larval *H. illucens*

In addition, RNA-seq data from the fat body of larval fifth-instar *H. Illucens* were analyzed. Of the 296 proteins identified in the hemolymph, the corresponding genes for 291 proteins were also expressed in the fat body (read count > 5 in at least three distinct samples).

We further analyzed the 1,000 most highly expressed genes in the fat body for functional annotation and focused on tissue-specific terms (Supplementary Table S2). Complete results including all housekeeping-related processes are available in Supplementary Table S3. Consistent with the hemolymph findings, the GO term “sensory perception of smell” was significantly enriched (FDR < 0.001), featuring 18 genes primarily encoding general odorant-binding proteins (Supplementary Tables S2 and S3).

The term “nutrient reservoir activity” was overrepresented (FDR < 0.001) and included eight genes encoding storage proteins: three hexamerin-1.1-like, two larval serum protein 1 alpha chain-like, one larval serum protein 1 gamma chain-like, and two larval serum protein 2-like variants (Supplementary Tables S2 and S3). Only the two larval serum proteins 2-like proteins were identified in the hemolymph (Supplementary Table S1, Fig. 1B). Multiple sequence alignment analysis of the eight predicted storage proteins revealed considerable sequence variation across the protein family (Supplementary Fig. S1).

Interestingly, the term “structural constituent of chitin-based larval cuticle” was significantly enriched among the most highly expressed genes in the fat body (FDR < 0.001), encompassing 21 genes, predominantly encoding different cuticle proteins (Supplementary Tables S2 and S3). One of these, flexible cuticle protein 12-like, was also identified in the hemolymph (Supplementary Table S1). Nine other genes with chitin-binding domains were also detected in the hemolymph, including chitinase and peritrophin proteins (Supplementary Table S1).

The Kyoto Encyclopedia of Genes and Genomes pathways for fatty acid metabolism and glycolysis/gluconeogenesis were enriched (FDR < 0.05), encompassing 16 and 15 genes, respectively (Supplementary Tables S2 and S3). Lipid storage-related genes, including lipid storage droplet surface-binding protein 1, lipid storage droplet surface-binding protein 2, and lipid droplet-associated hydrolase, were also among the top 1,000 most highly expressed genes in the fat body (Supplementary Tables S2 and S3).

### Gene Regulation in the Fat Body of Larval *H. illucens* Following Exposure to the Entomopathogenic Fungus *B. bassiana*

**Fig. 2.**
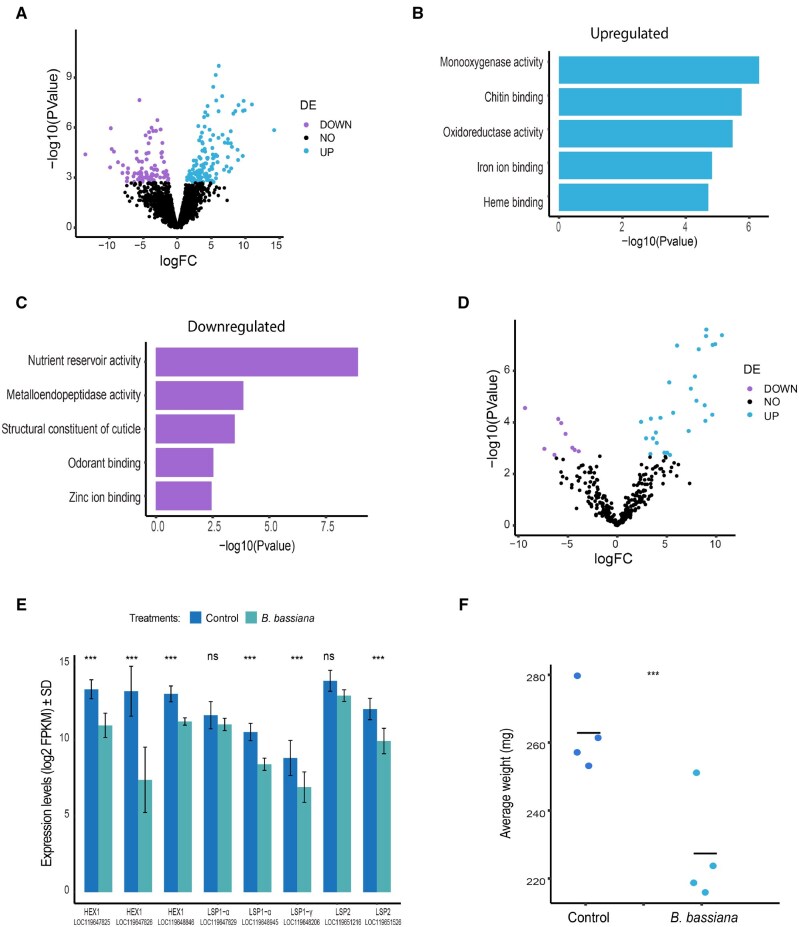
Regulation of gene expression in the fat body of larval *Hermetia illucens* following exposure to the entomopathogenic fungus, *Beauveria bassiana*. A) Volcano plots presenting -log10(*P*-values) versus log2(fold change) of gene expression for all expressed genes in the *B. bassiana* treatment versus the control. Black dots represent genes that did not meet the significance threshold (FDR < 0.1 and absolute fold-change > 1.5). Light blue dots denote upregulated genes (FDR < 0.1 and fold-change > 1.5), while purple dots highlight downregulated genes (FDR < 0.1 and fold-change < −1.5). B and C) Gene ontology enrichment analysis focused on the molecular functions of genes that were upregulated B) or downregulated C) in *B. bassiana* treatment versus the control (FDR < 0.05). D) Volcano plots presenting -log10(*P*-values) versus log2 (fold change) of gene expression for genes associated with cuticle and chitin-binding in the *B. bassiana* treatment versus the control. Black dots represent genes that did not meet the significance threshold (FDR < 0.1 and absolute fold-change > 1.5). Light blue dots denote upregulated genes (FDR < 0.1 and fold-change > 1.5), while purple dots highlight downregulated genes (FDR < 0.1 and fold-change < −1.5). E) Average expression levels (log2 FPKM) of genes associated with the term “nutrient reservoir activity” in control and *B. bassiana* treatments. Error bars represent standard deviation (SD). *** indicates FDR < 0.1. F) Average weight (mg) of larvae raised on a standard diet supplemented with *B. bassiana* or without it (control). Each dot represents a biological replicate. *** indicates *t*-test: *P* < 0.05. HEX1, hexamerin-1.1-like; LSP1-α, larval serum protein 1 alpha chain-like; LSP1-γ, larval serum protein 1 gamma chain-like; LSP2, larval serum protein 2-like; ns, nonsignificant.

To study how pathogen exposure modulates the expression of genes related to nutrient reservoirs and metabolism, we analyzed RNA-seq data from larval fifth instars of *H. illucens* raised on a standard diet supplemented with the entomopathogenic fungus *B. bassiana*, alongside a control group. Differential expression analysis between the *B. bassiana* and control groups revealed 215 significantly differentially expressed genes (FDR < 0.1 and absolute fold-change > 1.5), with a higher number of upregulated genes (2-sided exact binomial test: *k *= 126, *n *= 215, *P *= 0.01). The estimated probability of success was 0.586 (95% CI: [0.517, 0.653]; Fig. 2A).

Functional annotation revealed enrichment of molecular functions related to the cuticle within the differentially expressed genes. Among the upregulated genes, the term “chitin binding” was significantly enriched (FDR < 0.001; Fig. 2B), whereas the term “structural constituent of cuticle” was predominantly represented in the downregulated genes (FDR < 0.01; Fig. 2C). The majority of genes associated with the cuticle and chitin binding were upregulated (2-sided exact binomial test: *k *= 27, *n *= 36, *P *= 0.004). The estimated probability of success was 0.75 (95% CI: [0.578, 0.879]; Fig. 2D).

The term “nutrient reservoir activity” was enriched among the downregulated genes (Fig. 2C). To explore this further, we examined the eight genes encoding the storage proteins hexamerin-1-1 and larval serum proteins, which were highly expressed in the fat body and associated with the term “nutrient reservoir activity” (Supplementary Table S2). Six of them were significantly downregulated (FDR < 0.1; Fig. 2E). These results align with our findings, showing that larval *H. illucens* exposed to *B. bassiana* exhibit a reduction in body weight compared to the control group (t-test: *t* = −3.55, df = 6, *P *= 0.01; Fig. 2F). However, the expression levels of genes associated with other metabolic aspects, such as lipid storage, fatty acid metabolism, and gluconeogenesis, were not significantly altered.

Several odorant binding/sensory perceptions of smell associated genes, which were dominant in both the hemolymph and fat body tissues, were downregulated under *B. bassiana* treatment as well (Fig. 2C). Metalloendopeptidase activity and zinc ion binding related genes were also downregulated (Fig. 2C), while monooxygenase activity, oxidoreductase activity, iron ion binding, and heme binding associated genes were upregulated (Fig. 2B).

## Discussion

This study highlights the metabolic and immune interactions within the hemolymph and fat body of larval *H. illucens*, particularly in response to pathogen exposure. By integrating proteomic and transcriptomic analyses, we provide a comprehensive view of nutrient storage, metabolism, and immune modulation in this significant species. However, additional analyses, such as the evaluation of additional immune-related genes and broader nutritional parameters, would be necessary understand the full spectrum of immune pathways and its nutritional aspect.

Our proteomic analysis revealed a diverse array of proteins in the hemolymph, most of which were also expressed in the fat body. Notably, odorant-binding-like proteins were enriched in the hemolymph and expressed in the fat body. Odorant-binding proteins are known for their expression in the insect olfactory system and their ability to bind odorants (Sun et al. 2018, Park et al. 2024). However, growing evidence suggests that this protein family is involved in diverse chemo-sensing responses to exogenous stimuli, such as infection and stress (Benoit et al. 2017, Sun et al. 2018, Zhang et al. 2023). Certain olfactory proteins have been found to be expressed not only in olfactory tissues but also in insect immune tissues, such as hemocytes and fat bodies (Thomas et al. 2016, Zhang et al. 2023). For example, the expression of locust odorant-binding protein 11 was shown to be induced by infection with the entomopathogenic fungus, *Metarhizium anisopliae* and was found to participate in the detection and avoidance of the fungal-produced volatile compound, phenylethyl alcohol, but ultimately suppresses the immune responses to facilitate mycosis (Zhang et al. 2023). Interestingly, several genes encoding odorant-binding-like proteins were significantly downregulated in the fat body of larvae treated with *B. bassiana*. This observation suggests that odorant-binding-like proteins may play a role in the immune response of *H. illucens* larvae to fungal exposure.

Cuticle-related molecular functions were among the most highly expressed genes in the fat body, with chitin-binding proteins detected in the hemolymph. The function of cuticle genes in both the hemolymph and the fat body has previously been demonstrated, emphasizing their role in blood coagulation and immune responses to pathogenic infections (Liang et al. 2015, Zhao et al. 2017, Mallick and Eleftherianos 2024). For example, the transglutaminase enzyme group in *Drosophila melanogaster* is involved in cuticle morphogenesis, trapping invading pathogens, and hemolymph clotting (Wang et al. 2010, Shibata and Kawabata 2018). Additionally, the cuticular protein BmCPT1 is expressed in the fat body and hemocytes of silkworms and participates in the recognition of *Escherichia coli* (Liang et al. 2015). We found significant upregulation of cuticle-related genes in response to *B. bassiana* treatment. Hence, our findings further support a potential role for cuticle proteins in immune defense mechanisms in response to pathogens.

It was previously found that *H. illuces* exposure to B. bassiana, caused significant changes in the expression of antimicrobial peptides (Herman et al. 2024). The notable changes presented here in the expression of cuticle-related genes and odorant-binding-like proteins highlight their potential roles in immune defense and adaptation to stress. However, further research is needed to identify the key factors that regulate these processes and the connection between them.

As expected, among the most highly expressed genes in the fat body, those related to metabolic processes such as lipid storage, protein storage, fatty acid metabolism, and gluconeogenesis were dominant. Interestingly, eight different storage proteins (hexamerin/larval serum protein) encoding genes were expressed in the fat body, but only two larval serum protein 2-like variants were identified in the hemolymph. This could be explained by the limitations of the LC–MS/MS analysis method but may also indicate a real difference in the function of the various storage proteins, which possess essential sequence differences. It has been demonstrated that storage proteins are recaptured from the hemolymph by the fat body at different rates, with degradation and use varying by stage and sex (Telfer and Kunkel 1991, Burmester 2013).

The results also revealed a significant decrease in the expression levels of these storage protein-encoding genes in *H. illucens* larvae following *B. bassiana* exposure. This aligns with previous knowledge that *B. bassiana* pathogenicity involves nutrient uptake from the host body (Boucias and Pendland 1984, Pedrini et al. 2013, Wang et al. 2021), which causes starvation and reduction in the expression of storage proteins (Webb and Riddiford 1988, Yan et al. 2022). This may explain the observed decrease in larval body weight. Additionally, less energy is likely reserved for the non-feeding pupal stage, which could account for the impaired adult emergence previously reported in *H. illucens* larvae treated with *B. bassiana* (Mani et al. 2023).

In this study, we presented a diverse array of proteins from hemolymph and RNA expression profiles associated with the fat body of *H. illucens* larvae. Understanding these core tissues, which are integral to energy storage and metabolism, is crucial, particularly in *H. illucens* larvae. Our findings provide deeper insights into the physiological mechanisms underlying the response of *H. illucens* to pathogens, paving the way for further research into the functional roles of these pathways. Additionally, the results emphasize the critical role of storage proteins in the metabolic and developmental processes of *H. illucens*, while highlighting their susceptibility to pathogen-induced disruptions.

## Supplementary Material

ieaf074_Supplementary_Data

## Data Availability

RNA-seq data used in this article were obtained from a previous study (Herman et al. 2024) and are available in the NCBI GEO database GSE272202. LC/MS-MS datasets from this study are available upon request from the corresponding author.
