# Supplementary material for: Metabolic and immune functions of the hemolymph and fat body in Hermetia illucens (Diptera: Stratiomyidae) under pathogen challenge
Source: J Insect Sci. 2025 Oct 11;25(5):ieaf074. doi: 10.1093/jisesa/ieaf074 (PMC12513866; doi:10.1093/jisesa/ieaf074)
Supplement: ieaf074_Supplementary_Data [file ieaf074_supplementary_data.zip › Supplementary_fig1.pdf]

```
XP 037904991.1 hexamerin-1.1-like Hermetia illucens                         .MKSLVVLAFCLASAAAVLD...SVWDQLHGYG.VGGARTGWTGGRLMGDT...G   47
XP 037906648.1 hexamerin-1.1-like Hermetia illucens                         .MKLLVFLAFCLALVAGSVD...SLWGRLQGLDRVG...TGWTGQKLMGDT...G   45
XP 037904992.1 hexamerin-1.1-like Hermetia illucens                         .MRSLLVLAFCIALAAATVD...SLWGRLRGVGGLG...TGWAGGRLMGDT...G   45
XP 037906789.1 larval serum protein 1 alpha chain-like Hermetia illucens    .MKVLVLLFALVAFASAWLGRDVNDWKRDRYLYDVG..LGDHEYGKAWGDTVDYG   52
XP 037905738.1 larval serum protein 1 gamma chain-like Hermetia illucens    .MRILFVFLGGLALSAGFGP......NSRGDIYRGG.....YGYN..........   33
XP 037904997.1 larval serum protein 1 alpha chain-like Hermetia illucens    .MKIALVFMAILAICAG......................................   16
XP 037911086.1 larval serum protein 2-like Hermetia illucens                MKPIIVVLLACLVALVSS.....................................   18
XP 037910598.1 larval serum protein 2-like Hermetia illucens                MRSITVILLASFAILAS......................................   17
consensus                                                                    ******* ****  *         *         *            ***   *

XP 037904991.1 hexamerin-1.1-like Hermetia illucens                         LDDDVH.FSKYR.NLYDVG.......TVDTVDKLGWG....LHTGYPGIGKVG..   87
XP 037906648.1 hexamerin-1.1-like Hermetia illucens                         MDDDLY.LAKYG.KLSDVG.......MHDNMGPYGMG....MHSNMMDVSNMG..   85
XP 037904992.1 hexamerin-1.1-like Hermetia illucens                         IDDDIN.IGKYR.NLYDIG.......TADTVDKLGIGTGTGIHDDLIDLGRGD..   89
XP 037906789.1 larval serum protein 1 alpha chain-like Hermetia illucens    VNKHIQNFDDSREKLFGVGVDTDNKYTHHPLANILRGNTQESIENIYDDTHVKDR  107
XP 037905738.1 larval serum protein 1 gamma chain-like Hermetia illucens    .DMTLNYLGMDR.NAMNYD.........QNLD..........QAMLLDKNVMN..   65
XP 037904997.1 larval serum protein 1 alpha chain-like Hermetia illucens    .......................................................   16
XP 037911086.1 larval serum protein 2-like Hermetia illucens                .......................................................   18
XP 037910598.1 larval serum protein 2-like Hermetia illucens                .......................................................   17
consensus                                                                    *         *  *   *                 *          *

XP 037904991.1 hexamerin-1.1-like Hermetia illucens                         .......LKTVDDVRTVDNMDKKYVKYDDDVKLKILGDKHLLLKQKFILDVLMDV  135
XP 037906648.1 hexamerin-1.1-like Hermetia illucens                         .......LNTLDKVQTVDNVDKKLLKYDDDVKLKILGDKHLLLKQKFILDVLLDL  133
XP 037904992.1 hexamerin-1.1-like Hermetia illucens                         .......LRNLDRARTVDNVDKKLVRFDDDVKLKILGDKHLLIKQKFILDVLLNL  137
XP 037906789.1 larval serum protein 1 alpha chain-like Hermetia illucens    LYDIDHTYKTYGRVKTVPNVDKKLVKYDDDLKARILGDKDLLIKQKLILDVLRNV  162
XP 037905738.1 larval serum protein 1 gamma chain-like Hermetia illucens    ......RVDTSRNILTRDRLNIDQMGMGRDLKISIMNDKDILRKQKFILDIMRNV  114
XP 037904997.1 larval serum protein 1 alpha chain-like Hermetia illucens    ......................HAIRLKDDFGTKIDS.KDFLLKQKFIFDVLYHL   48
XP 037911086.1 larval serum protein 2-like Hermetia illucens                ..........................ASYMNRNVKYAETEFLEKQQFVFDILQHV   47
XP 037910598.1 larval serum protein 2-like Hermetia illucens                ..........................GSYMTKNVKYADKDFLEKQKFIFAMVQHP   46
consensus                                                                             *     **** ***    *** * ********! !!*******  *

XP 037904991.1 hexamerin-1.1-like Hermetia illucens                         HKPLHVDDVTH...NVHIVEEEIYYKNFDKVIDFFRLLKNKNVILPRGTVFTVTN  187
XP 037906648.1 hexamerin-1.1-like Hermetia illucens                         QKPMMLDHMID...NVHLVDGEIYYKNFDKVIDFFRMLKNKEVILPRGVPFTVTN  185
XP 037904992.1 hexamerin-1.1-like Hermetia illucens                         HKPMR.DDTTD...VVHIVDGEIYYKNFDKVIDFFRLLTNRSTLLPRGAVFTITN  188
XP 037906789.1 larval serum protein 1 alpha chain-like Hermetia illucens    YATWTTDEKDKD..FARLVEGEVNYKNFDKVIDFFHLLKNKNVLLPRGQIFTIKN  215
XP 037905738.1 larval serum protein 1 gamma chain-like Hermetia illucens    YTTLIMDDVRD....VAPIEGEMNYNNYDRVIPFFRMYG.MKMFLPRGRIFAVSD  164
XP 037904997.1 larval serum protein 1 alpha chain-like Hermetia illucens    HMPLGEEYVKD...VHGFIEGKEYYNDYDKVVKFFMMYK..YGLLPRNFVFSIFN   98
XP 037911086.1 larval serum protein 2-like Hermetia illucens                HQNVVLLPKYKDGKDFDIQNYYDHYNHVELVKEFYEWHT..KSPVPVGEIFTLFN  100
XP 037910598.1 larval serum protein 2-like Hermetia illucens                FQDDIYEKKYMDGKSFKLEDHFDHFEDVEKVKLFWDMYL..HDPVPPGEILTLFT   99
consensus                                                                   * *   *   *    *  **** *******!**!******    *!** ***  *

XP 037904991.1 hexamerin-1.1-like Hermetia illucens                         KLHRNQMKLLFHLFYYSKDFIVFQKNVLWARQFINEELFVHALIAAVLHRDDMVG  242
XP 037906648.1 hexamerin-1.1-like Hermetia illucens                         KLHRKQMKLLFHLFFYSKDFLVFQKNVLWARKFINEEMFVHALVAAVLHRDDMIG  240
XP 037904992.1 hexamerin-1.1-like Hermetia illucens                         KVHRRQLRLLFNLFVYSKDFLVLQKNVLWARRNINEEMFVHALIAAVLHRDDMVG  243
XP 037906789.1 larval serum protein 1 alpha chain-like Hermetia illucens    KIHVKQLRLLFNLFVYSKDFKVFRMNVLWARKYVNEEMFAYALIAAVLHRDDLVG  270
XP 037905738.1 larval serum protein 1 gamma chain-like Hermetia illucens    RVQLRQAVLLFNLFYYAKDFNIFQRNVLWARNNVNEDMFIYSLTAAIFQRRDMIG  219
XP 037904997.1 larval serum protein 1 alpha chain-like Hermetia illucens    YRHITEMKGLFYLFYYSRDWPTFYHNVLYARQFVNEGMFVYALYAAVIHRKDLQG  153
XP 037911086.1 larval serum protein 2-like Hermetia illucens                EHHVEYAKRLVYAFVLAKDWDTFYKTVVWARWHVNKEVFVYALSIAVIHRTDLQG  155
XP 037910598.1 larval serum protein 2-like Hermetia illucens                THHYTYVKYLSYLFLYAKDWDTFYKTVLWARFHVNKAVFIYALTFAVTHRSDMQG  154
consensus                                                                   * *  * **!* *! ***!* *****!**!!  *!***!***! *!***!*!* !
```

```
XP 037904991.1 hexamerin-1.1-like Hermetia illucens                      VKVPALYEILPYKFHTSMTIDKALKLKDITVVDKK......DLVIFNKYTKDYMT   291
XP 037906648.1 hexamerin-1.1-like Hermetia illucens                      VKLPALYEILPYKFHTSMTIDKALKLKDV.VMDKK......DLIIFNKYTKDYMM   288
XP 037904992.1 hexamerin-1.1-like Hermetia illucens                      VKLPALYEILPHKFHTSMTIDKALKLKDITRLDKK......DLVIWNKHTKDHML   292
XP 037906789.1 larval serum protein 1 alpha chain-like Hermetia illucens  VKLPAIYEIFPYKFHTSVTIDKALKLKDDLIFRNK......DLIVLNKYTSDYVT   319
XP 037905738.1 larval serum protein 1 gamma chain-like Hermetia illucens  IKLPSHYEVMPHKFHTALTITNAVNTRMG.NLQDK......VLIVKNNYTSDITL   267
XP 037904997.1 larval serum protein 1 alpha chain-like Hermetia illucens  IILPAHYEVLPYMYFNGLTIDEIMKYKTLGFSKMEN.....DVVILSKYTDDINI   203
XP 037911086.1 larval serum protein 2-like Hermetia illucens              LVLPAIYEIFPYYFFNSETIQKAQHYKMQGFDGVKKVEGVYNVVIPSNYSGVHGQ   210
XP 037910598.1 larval serum protein 2-like Hermetia illucens              FVLPAIYEIYPQLFFNAETIQKAQHYKMQGFHGVKLVENVYNVVIPVNYTGVHGH   209
consensus                                                                ***!* !!**!****** !!********      *      **** **** *

XP 037904991.1 hexamerin-1.1-like Hermetia illucens                      MNDEGKLAYLMEDIGWNLYHYFMYMDFASWIGGK.DFNLFKERRGEKYLYFIQQI   345
XP 037906648.1 hexamerin-1.1-like Hermetia illucens                      MNDEGKLAYLMEDIGWNLYNFFFHMEFGAWIGGK.DFNLFKERRGEKYLYFLHQI   342
XP 037904992.1 hexamerin-1.1-like Hermetia illucens                      MNDEGRLAYLTEDIGWNLYHFFFHMDFASWIGGK.DFNLFKERRGEKYLYFLQQI   346
XP 037906789.1 larval serum protein 1 alpha chain-like Hermetia illucens  LNDEGKLTYLTEDIGWNMYNFFFHMEFASWIGGK.DFKLDIERRGEKYLYHIQQI   373
XP 037905738.1 larval serum protein 1 gamma chain-like Hermetia illucens  MNDEAKLAYFTEDIGLNLYHYYLFLQYPYWLGGE.EFGLYKQMRGEGYLLLIQQI   321
XP 037904997.1 larval serum protein 1 alpha chain-like Hermetia illucens  MNEESKLSYFTEDIGWNMYYYFFNLDYPYWLGGK.DFNLYKDKRGEQFLYMHQQL   257
XP 037911086.1 larval serum protein 2-like Hermetia illucens              VNDENLVSYFTEDIGLNAFYQYYNLDYPYWTSGAVKGDFVKDRHGEFYFSVHQQL   265
XP 037910598.1 larval serum protein 2-like Hermetia illucens              VNKENKLSYFTEDIGLNAFYADYNKDYAFWTKGKVDGDFIKDRRGEFWLNLHQQI   264
consensus                                                                *!*!****! *!!!!*!**  ** ** * !**!* **** ****!!****  *!*

XP 037904991.1 hexamerin-1.1-like Hermetia illucens                      LARYNLERLSNGLGHIKDISLWDYIKTGYNPMMVYKNGLPVTVRKNYYDIRETGD   400
XP 037906648.1 hexamerin-1.1-like Hermetia illucens                      LARYNLERLSNGLGNIKDISLWDYIKTGYNPMMIQKNGLPVTMRKNYYDVRETGD   397
XP 037904992.1 hexamerin-1.1-like Hermetia illucens                      LARYNLERISNGLGNIKDITLWDYIKTGYNPMMVQKNGLPVTMRKNYYDIRETGD   401
XP 037906789.1 larval serum protein 1 alpha chain-like Hermetia illucens  LARYYLERISHGLGDVKDILQWGVVKDGYNPLLTSFNGLPFTSRKNYYDISSTGN   428
XP 037905738.1 larval serum protein 1 gamma chain-like Hermetia illucens  LARYNMERLSHGMGPIKDISLNIPLKNGYNSFLRHYNGLGYSVRKNYYDVYTTGD   376
XP 037904997.1 larval serum protein 1 alpha chain-like Hermetia illucens  LARYYLERLSNKMGDIPEFSWYSKIKTGYDPHMKYYNGLPFTVRKNYYETFTTGD   312
XP 037911086.1 larval serum protein 2-like Hermetia illucens              LARYYMERLSNSLTDIEEFNFNTETFPGYFSGLRYYSGVHFPDRENGYGFYHSGN   320
XP 037910598.1 larval serum protein 2-like Hermetia illucens              LARYYMERLSNNLPEIEEFHWDIEHFPGYYPGLRYYGGVHFPDRENDYGFYHSGN   319
consensus                                                                !!!! *!!*!**** *******  ***!!**   ***!**** !*!*!*   *!*

XP 037904991.1 hexamerin-1.1-like Hermetia illucens                      YFLMKRVDDYARRIRDAIDQGYSITIDGGKVDLK.RSDAIDILGNMIQWNIDSVN   454
XP 037906648.1 hexamerin-1.1-like Hermetia illucens                      YFLMKRVNDFERRIRDAIDKGVCTTIDGGLVDLK.RSDAIDILGNMIQWNIDSVN   451
XP 037904992.1 hexamerin-1.1-like Hermetia illucens                      YFLMKRVGDFERRIRDAIDKGYCTTIDGGKVDLK.RSDAIDILGNMIQWNIDSVN   455
XP 037906789.1 larval serum protein 1 alpha chain-like Hermetia illucens  YELIDRIGDYGRRIRDAIDEGFCTATDGGIVDLK.RWDGADILGNLIQGNVDSVN   482
XP 037905738.1 larval serum protein 1 gamma chain-like Hermetia illucens  YYLISRVKDYERRIRDAIDKGYVIGKDGSKIDLY.GKNGIDLLGNIIQSNKDSVN   430
XP 037904997.1 larval serum protein 1 alpha chain-like Hermetia illucens  YYPLNRIEDFERRIRDAIDLGYIIGFDGAKIDLW.SKEGIDLLGNLIQSNPDSLN   366
XP 037911086.1 larval serum protein 2-like Hermetia illucens              FYNIDLVTLYSHRIYDVIDYNFALLPDGKHFELKDVKVALETLGNIVQGNKDSVN   375
XP 037910598.1 larval serum protein 2-like Hermetia illucens              YLYIDLAIMYSHRIMDVIDMNFAILPDGKHFPLDSVKVALETLGNLIQGNEDSVN   374
consensus                                                                * ** ** ****!!*!*!! ** *  !!****!* *******!!! *! ! !!*!

XP 037904991.1 hexamerin-1.1-like Hermetia illucens                      KRYYGSLIIDFVKLLGGGYVKNVDDFKLTPSVVDHYETALRDPVIYQLYKKIMKM   509
XP 037906648.1 hexamerin-1.1-like Hermetia illucens                      SRFYGSLLVDIFKLLGGGYVKNVDDFKLTPSVMDHYETALRDPVTYQIYKKVMKM   506
XP 037904992.1 hexamerin-1.1-like Hermetia illucens                      TRFYGSLIIDLLRLLGGGHVKNVDDFKLTPSVMDHHETALRDPVTYQLYKKILKI   510
XP 037906789.1 larval serum protein 1 alpha chain-like Hermetia illucens  TKLYGGFIKNFFKLLGSGDLKNVDDFKLTTSVLEHHETVLRDPVTYQILKKITKL   537
XP 037905738.1 larval serum protein 1 gamma chain-like Hermetia illucens  DRFYGGLVRLMRKLIGGGYVKNVDHNNVLPSVMEYYETALRDPATYQVYKRVLKL   485
XP 037904997.1 larval serum protein 1 alpha chain-like Hermetia illucens  EKYFGKLIAFAHKMLGGGKFYELFEYKVIPGAMEHYETMLRDPIFYQLYKRILKF   421
XP 037911086.1 larval serum protein 2-like Hermetia illucens              PQLYGSMDHILRDIINEG.FPAGHHGEDVPGVMYHYSTSIRDPAFFHMYKFILRF   429
XP 037910598.1 larval serum protein 2-like Hermetia illucens              RKLYGSMDWLLRTIINEG.FPTGVHGNEVPGVLYHYETSMRDPAFYFMYKYMMRF   428
consensus                                                                 * *!***    *****! ************** ***!**!!!**** *!****
```

```
XP 037904991.1 hexamerin-1.1-like Hermetia illucens                       VYMFKDYLPTYTKTDLLFDAVHVDNIRTTKLITYFDLFDVDLRTMINLDSLKTKD   564
XP 037906648.1 hexamerin-1.1-like Hermetia illucens                       VHMFKGYLPAYTKTDLLLDGVRVDNIRTSKFMTYFDLFDVDIRTMINLDDLKMKH   561
XP 037904992.1 hexamerin-1.1-like Hermetia illucens                       VRLFKENLPTYTKNELLLDGVRVDNIRTSKLLTYFDLFDVDIRTIVNLGDLKLRD   565
XP 037906789.1 larval serum protein 1 alpha chain-like Hermetia illucens  YYQLKDYLPSYKKDDLLLDGVRVVDVKTTPLVTYFDFFDVDITNIVGVDPLKYKT   592
XP 037905738.1 larval serum protein 1 gamma chain-like Hermetia illucens  LYQYKNTLRPYTTEDLLVKGVRIMDIRTTKLVTYCDQFDVDVSTIVDHN.IRTQT   539
XP 037904997.1 larval serum protein 1 alpha chain-like Hermetia illucens  WFQYKLKLPPYKYDDLLLPGVKINKVDISKLVTYFDEFDIDVSTLMEVDKIPMDY   476
XP 037911086.1 larval serum protein 2-like Hermetia illucens              YWRYLDRLPSYTKSELQFDGVKITGVEMDKLVTFFDKFYADITNAVDVEPYDVSK   484
XP 037910598.1 larval serum protein 2-like Hermetia illucens              YWHYLNSLKPYTKEDLLFDGVKIESVEMDKLITYFDKFDADITNIVDVEPYDEKV   483
consensus                                                                    **  !* !** *!****!*    ** ***!**! !**!* *** ** ** *

XP 037904991.1 hexamerin-1.1-like Hermetia illucens                       VHLG....DDRVGKMDKLVV...................................   580
XP 037906648.1 hexamerin-1.1-like Hermetia illucens                       MDIS....DDRIGKMDKFVI...................................   577
XP 037904992.1 hexamerin-1.1-like Hermetia illucens                       AHIT....DDRIGRMDKLVV...................................   581
XP 037906789.1 larval serum protein 1 alpha chain-like Hermetia illucens  VDTVGGFDDVHKVTKDTIIR...................................   612
XP 037905738.1 larval serum protein 1 gamma chain-like Hermetia illucens  KDTN...VDNVVG..DKLVF...................................   554
XP 037904997.1 larval serum protein 1 alpha chain-like Hermetia illucens  FKKG..YKDVDYMSKDYIDMDYKFPYGGKFFDKDYIPDKYMDTDMKYDYKMNWKW   529
XP 037911086.1 larval serum protein 2-like Hermetia illucens              GTELYKFGRTASYDGHDFVIK..................................   505
XP 037910598.1 larval serum protein 2-like Hermetia illucens              STDLYKFGRVANWEGHDFVIK..................................   504
consensus                                                                         *   *  ** *

XP 037904991.1 hexamerin-1.1-like Hermetia illucens                       .........ARHMRLNHKPFTINIDLTVDRPEKVVIRTFLGPKFDADGKILKIKY   626
XP 037906648.1 hexamerin-1.1-like Hermetia illucens                       .........ARHMRLNHKPFTVNIDLTVDQPQKVVIRMFLGPKFDADGKIWKLKY   623
XP 037904992.1 hexamerin-1.1-like Hermetia illucens                       .........ARHMRLNHKPFTIDIDLTVDQPQKVVVRLFLGPKFDSDGRLIKLKN   627
XP 037906789.1 larval serum protein 1 alpha chain-like Hermetia illucens  .........ARHVRLNHKPFTITLDLNVDKPQKVVFRIFLGPKFDSDGKLLDLKK   658
XP 037905738.1 larval serum protein 1 gamma chain-like Hermetia illucens  .........ARHTRLNHKPFAINLDVTSDRAQKVVVRLFIGPKYDQFGKKIQLKD   600
XP 037904997.1 larval serum protein 1 alpha chain-like Hermetia illucens  GDSFNYKYKVRQYRLNHKPFKMTLDVASDKPQTVVFRIYMGPKFDSMGKLININD   584
XP 037911086.1 larval serum protein 2-like Hermetia illucens              .........ARQQRLNHLPFTFKLSVSSTKPQKAVVKVMIGPKYDVYGKKIDINV   551
XP 037910598.1 larval serum protein 2-like Hermetia illucens              .........ARQLRLNHLPFTYKVNVHSDKAHKAMVKVFIGPKFDFYGKKIDFAD   550
consensus                                                                          *!* !!!!*!!** ** * ********* **!!!*! *!* * **

XP 037904991.1 hexamerin-1.1-like Hermetia illucens                       NRDNFVELDKFTYDLTTVGKVTIKRDSRDFVYTVKDRTSIMDIYKLVLLNGDVNK   681
XP 037906648.1 hexamerin-1.1-like Hermetia illucens                       NRDNFVELDKFTYDLTNVGKVVIKRDSRDFVYTVKDRTSIMDIYKMVLLNNDVSK   678
XP 037904992.1 hexamerin-1.1-like Hermetia illucens                       NRRNFVELDKFTYDVAHVGKVTIKRDSRDFVYTVKDRTSIMDIYKIVLLNNDITK   682
XP 037906789.1 larval serum protein 1 alpha chain-like Hermetia illucens  NRKNFVEIDKFTRDLT.TGQVRIERNSRDLVYTVKDRTTFLDIYKKVLLNADVNS   712
XP 037905738.1 larval serum protein 1 gamma chain-like Hermetia illucens  NRDNFMELDKFVFDLP.AGRVTIKRDSRDFNYVVRDRTTYVDLYKNIVLNKDLNK   654
XP 037904997.1 larval serum protein 1 alpha chain-like Hermetia illucens  NRENFVEIDKFIYDLK.SGLNTVYRESREFYFSAKDRTMYSDLYKMVMMGLKG.K   637
XP 037911086.1 larval serum protein 2-like Hermetia illucens              NRKNFFELDYFLVDLV.EGENVITRSSTDYSWYVNDRTTYYELYKKLFQAVNSHE   605
XP 037910598.1 larval serum protein 2-like Hermetia illucens              NRKNFFEMDVFLVDLV.EGKNVITRSSNDFSWYVNDRTTYYELYKKLWQATTTDY   604
consensus                                                                 !! !!*!*!*!**!*   !*****!*!********!!!** * !! **** *  *

XP 037904991.1 hexamerin-1.1-like Hermetia illucens                       LVDMHVDEANCGFPDRLVLPKGWVGGMPMQLFVIVTKAKD...VVKRDFDHTIIC   733
XP 037906648.1 hexamerin-1.1-like Hermetia illucens                       LIDMHIDEANCGFPDRLILPKGWVGGMPMQLFVIVTKAKD...IVKRDLDHTLIC   730
XP 037904992.1 hexamerin-1.1-like Hermetia illucens                       LVDMHVDEANCGFPDRLVLPKGWVGGMPMQLFVIVTKAKD...IVKRDMDHTILC   734
XP 037906789.1 larval serum protein 1 alpha chain-like Hermetia illucens  VIGMHVDEANCGFPDRLLLPKGWIGGLPLQLFVIVTKAKD...VVKKDIHSSLMC   764
XP 037905738.1 larval serum protein 1 gamma chain-like Hermetia illucens  LLDLNIVESHCGFPDRLLLPRGWVGGMPLQIFAIITRATN...FGK.GPTILTKC   705
XP 037904997.1 larval serum protein 1 alpha chain-like Hermetia illucens  SFSFDIFEAHNGFPDRLLLPKGWVSGMPMQFFFIITPFKD...VKQYSKFWFSSY   689
XP 037911086.1 larval serum protein 2-like Hermetia illucens              KFPLDMSEAHCGFPSRLMLPKGKKGGMPFQFFFYV....................   640
XP 037910598.1 larval serum protein 2-like Hermetia illucens              KFPLDMSEAHCGFPNRLMLPKGKKGGMPFQFFFFITPYHEPEVKQFTGFDPTISC   659
consensus                                                                 * *** *!* *!!!*!! !!*!***!*!*!*!********    ** * * *  *
```

```
XP 037904991.1 hexamerin-1.1-like Hermetia illucens                  GKRTLINEVDDFSLGFPFDRRIDETEFYTKNMLFKDVLIFHDDDTNLVLNALDVG   788
XP 037906648.1 hexamerin-1.1-like Hermetia illucens                  GKRTLINEVDDYALGFPFDRRIDETEFYTKNMLFKDVLIFHDDDVNMMLNMMDVR   785
XP 037904992.1 hexamerin-1.1-like Hermetia illucens                  GKRTLINEVDDFALGFPFDRRIDETEFYTKNMLLKDVLIFHDDDTNMALKMLDIG   789
XP 037906789.1 larval serum protein 1 alpha chain-like Hermetia illucens   GKNTLINEVDDLALGFPFDRPIDLTEFYTKNMLFKDVLVFHDIGD..VTKVKDLD   817
XP 037905738.1 larval serum protein 1 gamma chain-like Hermetia illucens   GRNTQLFHVDDFKLGFPFDRYIDETEFYTTNMLFKDILIYHEGMDT.NIGMNDYN   759
XP 037904997.1 larval serum protein 1 alpha chain-like Hermetia illucens   .IGSGSRFFDDLPLGYPFDRLIDEGFFYTPNMYFKDVFIYHLDTIPKYSDFDSYK   743
XP 037911086.1 larval serum protein 2-like Hermetia illucens          .......................................................   640
XP 037910598.1 larval serum protein 2-like Hermetia illucens          GIGSGARYLSDLPFIYPLERKIDVKSFFVDNMYIYDTYIFHKSEADVNAIHY...   711
consensus                                                            ** ********  ******* **********************         *
```

```
XP 037904991.1 hexamerin-1.1-like Hermetia illucens                  GTVKDVGVLPRTVGVWDTHDTFADTRNTLTDTTNIMDMMRMKMTMV.........   834
XP 037906648.1 hexamerin-1.1-like Hermetia illucens                  GKMKDVGVMSKTMGVWDNHDTLMDTSN....KMNLMDMMRMKMTMV.........   827
XP 037904992.1 hexamerin-1.1-like Hermetia illucens                  GRGIDVGTVNKRVNVLDSHE.VMDTSD....KMRLMNIMKMTVV...........   828
XP 037906789.1 larval serum protein 1 alpha chain-like Hermetia illucens   GR...................VVDSKD.......FVDIDNIK.............   833
XP 037905738.1 larval serum protein 1 gamma chain-like Hermetia illucens   GMR....DITDTMGNLNPGLDVATIQN.IGGGRMMQDMMGDRMMRL.........   800
XP 037904997.1 larval serum protein 1 alpha chain-like Hermetia illucens   GLMYKLYPGMYNYKDLMMNKMDSGSYYKYPWMMDKMGMYDYKDMDYDYKLMGKKD   798
XP 037911086.1 larval serum protein 2-like Hermetia illucens          .......................................................   640
XP 037910598.1 larval serum protein 2-like Hermetia illucens          .......................................................   711
consensus                                                            *                      *           ****  *
```

```
XP 037904991.1 hexamerin-1.1-like Hermetia illucens                  .......................................................   834
XP 037906648.1 hexamerin-1.1-like Hermetia illucens                  .......................................................   827
XP 037904992.1 hexamerin-1.1-like Hermetia illucens                  .......................................................   828
XP 037906789.1 larval serum protein 1 alpha chain-like Hermetia illucens   .......................................................   833
XP 037905738.1 larval serum protein 1 gamma chain-like Hermetia illucens   .......................................................   800
XP 037904997.1 larval serum protein 1 alpha chain-like Hermetia illucens   YGMYDYKDLGYDYKPMDYGVSSYYKYPFGQYKDIDYDYDYKLTDKKDYISYYKYP   853
XP 037911086.1 larval serum protein 2-like Hermetia illucens          .......................................................   640
XP 037910598.1 larval serum protein 2-like Hermetia illucens          .......................................................   711
consensus
```

```
XP 037904991.1 hexamerin-1.1-like Hermetia illucens                  .......................................................   834
XP 037906648.1 hexamerin-1.1-like Hermetia illucens                  .......................................................   827
XP 037904992.1 hexamerin-1.1-like Hermetia illucens                  .......................................................   828
XP 037906789.1 larval serum protein 1 alpha chain-like Hermetia illucens   .......................................................   833
XP 037905738.1 larval serum protein 1 gamma chain-like Hermetia illucens   .......................................................   800
XP 037904997.1 larval serum protein 1 alpha chain-like Hermetia illucens   WVMDKFGTYKDMDYDYKTFDNKDYGTYYKYPYSPDKFGSYKDIDYDYKLLDKKDY   908
XP 037911086.1 larval serum protein 2-like Hermetia illucens          .......................................................   640
XP 037910598.1 larval serum protein 2-like Hermetia illucens          .......................................................   711
consensus
```

```
XP 037904991.1 hexamerin-1.1-like Hermetia illucens                  .......................................................   834
XP 037906648.1 hexamerin-1.1-like Hermetia illucens                  .......................................................   827
XP 037904992.1 hexamerin-1.1-like Hermetia illucens                  .......................................................   828
XP 037906789.1 larval serum protein 1 alpha chain-like Hermetia illucens   .......................................................   833
XP 037905738.1 larval serum protein 1 gamma chain-like Hermetia illucens   .......................................................   800
XP 037904997.1 larval serum protein 1 alpha chain-like Hermetia illucens   ISYYDYPYSPDKYGVYKDMDYDYKTFDKKDYGSYYKYPYSLDKFGVYKDMDYDYK   963
XP 037911086.1 larval serum protein 2-like Hermetia illucens          .......................................................   640
XP 037910598.1 larval serum protein 2-like Hermetia illucens          .......................................................   711
consensus
```

```
XP 037904991.1 hexamerin-1.1-like Hermetia illucens                         .......................................................  834
XP 037906648.1 hexamerin-1.1-like Hermetia illucens                         .......................................................  827
XP 037904992.1 hexamerin-1.1-like Hermetia illucens                         .......................................................  828
XP 037906789.1 larval serum protein 1 alpha chain-like Hermetia illucens    .......................................................  833
XP 037905738.1 larval serum protein 1 gamma chain-like Hermetia illucens    .......................................................  800
XP 037904997.1 larval serum protein 1 alpha chain-like Hermetia illucens    TFNKKDYGSYYKYPYSLDKFGVYKDMDYDYKIFDKKDYGSYYKYPYSLDKFGVYK  1018
XP 037911086.1 larval serum protein 2-like Hermetia illucens                 .......................................................  640
XP 037910598.1 larval serum protein 2-like Hermetia illucens                 .......................................................  711
consensus

XP 037904991.1 hexamerin-1.1-like Hermetia illucens                         .......................................................  834
XP 037906648.1 hexamerin-1.1-like Hermetia illucens                         .......................................................  827
XP 037904992.1 hexamerin-1.1-like Hermetia illucens                         .......................................................  828
XP 037906789.1 larval serum protein 1 alpha chain-like Hermetia illucens    .......................................................  833
XP 037905738.1 larval serum protein 1 gamma chain-like Hermetia illucens    .......................................................  800
XP 037904997.1 larval serum protein 1 alpha chain-like Hermetia illucens    DMDYDYKTFDKKDYGSYYKYPYSLDKFGGYKDMDYDYKLLDKKDYISYYQYPWMT  1073
XP 037911086.1 larval serum protein 2-like Hermetia illucens                 .......................................................  640
XP 037910598.1 larval serum protein 2-like Hermetia illucens                 .......................................................  711
consensus

XP 037904991.1 hexamerin-1.1-like Hermetia illucens                         .......................................................  834
XP 037906648.1 hexamerin-1.1-like Hermetia illucens                         .......................................................  827
XP 037904992.1 hexamerin-1.1-like Hermetia illucens                         .......................................................  828
XP 037906789.1 larval serum protein 1 alpha chain-like Hermetia illucens    .......................................................  833
XP 037905738.1 larval serum protein 1 gamma chain-like Hermetia illucens    .......................................................  800
XP 037904997.1 larval serum protein 1 alpha chain-like Hermetia illucens    DKSSMYDYKDIDYDYYKTFDKFGSGMYHKYPWMMKTMYKPDSFYTSYNYPEVMFE  1128
XP 037911086.1 larval serum protein 2-like Hermetia illucens                 .......................................................  640
XP 037910598.1 larval serum protein 2-like Hermetia illucens                 .......................................................  711
consensus

XP 037904991.1 hexamerin-1.1-like Hermetia illucens                         .....   834
XP 037906648.1 hexamerin-1.1-like Hermetia illucens                         .....   827
XP 037904992.1 hexamerin-1.1-like Hermetia illucens                         .....   828
XP 037906789.1 larval serum protein 1 alpha chain-like Hermetia illucens    .....   833
XP 037905738.1 larval serum protein 1 gamma chain-like Hermetia illucens    .....   800
XP 037904997.1 larval serum protein 1 alpha chain-like Hermetia illucens    SIYKK  1133
XP 037911086.1 larval serum protein 2-like Hermetia illucens                 .....   640
XP 037910598.1 larval serum protein 2-like Hermetia illucens                 .....   711
consensus
```

X non-conserved

X ≥50% conserved

Supplementary figure 1: Multiple sequence alignment of the storage proteins

Multiple sequence alignment of the eight predicted storage proteins was performed using the msa R package (Bodenhofer et al. 2015). Protein names are indicated next to each sequence

References

Bodenhofer U, Bonatesta E, Horejš-Kainrath C, et al. 2015. msa: an R package for multiple sequence alignment. Bioinformatics. 31:3997–3999. https://doi.org/10.1093/bioinformatics/btv494
